# What Are the Real-World Podcast-Listening Habits of Medical Professionals?

**DOI:** 10.7759/cureus.16240

**Published:** 2021-07-07

**Authors:** Damian Roland, Brent Thoma, Andrew Tagg, Jason Woods, Teresa M Chan, Jeffrey Riddell

**Affiliations:** 1 Children's Emergency Medicine, Leicester University and Hospitals, Leicester, GBR; 2 Emergency Medicine, University of Saskatchewan, Saskatoon, CAN; 3 Emergency Medicine, Footscray Hospital, University of Melbourne, Melbourne, AUS; 4 Emergency Medicine, University of Colorado School of Medicine, Aurora, USA; 5 Emergency Medicine, McMaster University, Hamilton, CAN; 6 Emergency Medicine, University of Southern California Keck School of Medicine, Los Angeles, USA

**Keywords:** podcasts, medical education, listening habits, playback speed, multitasking

## Abstract

Introduction

Educational podcasts are increasingly being utilized by health professionals for continuing education, but how they are being used remains poorly understood. Given their extensive reach, they represent a phenomenal opportunity for researchers to engage in knowledge translation of their scholarly works. The design, study, and effectiveness of these resources should be informed by a deeper understanding of their pragmatic usage. We aimed to prospectively determine the pragmatic, real-world listening habits of health professionals.

Methods

We performed a prospective observational study of a broad, interprofessional sample of participants (medical students, residents, physicians, nurses, physician assistants, and paramedics) recruited through a multimodal social media (Twitter and Facebook) campaign. Recruitment materials included an infographic and study website. Participants listened to eight podcasts and described their use of each in subsequent questionnaires.

Results

A total of 393 participants enrolled in the study, and 241 completed the survey for all eight podcasts. Listening behaviors were consistent across the podcasts with the majority selecting a normal speed of playback and engaging in concomitant activities such as driving. One-third of participants paused the podcasts due to interruption.

Conclusion

We describe the prospective use of medical podcasts by a cohort of health professionals. This work should inform the role of podcasts in the communication of medical research.

## Introduction

There is increasing evidence that methods for engaging in knowledge translation and, hence, education of frontline health professionals are changing [1]. Podcasts are playing an increasing role in the post-publication discussion of medical research and as a source of asynchronous learning for clinicians, who use them to both facilitate and supplement learning [2-4]. However, relatively little is known about how they are being used. Previous literature has found that many clinicians report listening while conducting other tasks [5]. This "multitasking" may result in only partial attention to the podcast, suggesting that clinicians’ listening habits may not be fully aligned with their stated goals of learning [6,7]. The disconnect between reported listening habits and optimal learning behaviors may be significant, given the reported impact of podcasts on clinical decisions, and may decrease their potential as knowledge translation vehicles [4].

The literature on podcast-listening habits is largely retrospective and provides little granular detail to guide podcast utilization or further study [4,6,7]. Further, because podcasts are complexly interwoven [6] with individual learners and their habits; literature on other media may not translate to podcasts. We sought to provide a richer understanding of how listeners use podcast technology by investigating the pragmatic, real-world listening habits of health professionals.

## Materials and methods

We performed a prospective survey-based study of the listening habits of an interprofessional sample of emergency medicine and critical care clinicians. Study participants listened to eight emergency medicine and critical care podcasts and completed podcast-specific surveys after each. The study was deemed exempt from ethical review (Research Ethics Board at the University of Saskatchewan, BEH 17-170).

Participant recruitment

We recruited participants through the METRIQ (Medical Education Translational Resources: Impact and Quality) methodology as described in a previous publication [7]. Briefly, we shared information, predominately via Facebook and Twitter, about the study to a targeted virtual community of practice that utilizes Free Open Access Medical education resources such as podcasts [7]. We also reached out via email to our personal networks and collaborators from previous studies [8-10]. Interested participants submitted an intake form requesting further information and were considered formally enrolled after completing an initial survey containing a standard consent form [7]. To incentivize participation and acknowledge the extensive time commitment of this study, we recognized participants who completed all surveys as contributors.

Survey design and podcast selection

We selected podcasts listed on the Social Media Index [10-12] for inclusion in the study. Eight were selected because we felt that this was the maximum number that participants would be willing to complete. To increase the generalizability of our results, we ensured variety in podcaster accent and geographic origins (two each with native speakers from Canada, the United States, the United Kingdom, and Australia) as well as a number of podcasters (four with a single podcaster and four with multiple). In keeping with previous research about ideal podcast episode length [4], all selected podcasts were 17-23 minutes in length (Table 1). Preference was given to recently published podcasts to ensure relevance and decrease the likelihood of being previously heard by the participants. The owners of the eight podcasts provided consent for their use in the study. To encourage the participants to listen to the podcasts in their usual fashion, a dedicated podcast channel was created via Soundcloud.com that could be added to any podcast application or accessed via any web browser.

**Table 1:** Characteristics of the selected podcasts

Podcast	Country of origin	Number of speakers	Length (minutes)
Broomedocs	Australia	Multiple	23
Royal College of Emergency Medicine (United Kingdom)	United Kingdom	Single	23
CRACKCast	Canada	Multiple	22
EMCrit	United States	Single	23
FOAMcast	United States	Multiple	23
PHARM Podcast	Australia	Single	21
Skeptic’s Guide to Emergency Medicine	Canada	Single	17
Resus Room	United Kingdom	Multiple	21

Each podcast-specific survey questionnaire investigated the real-world habits of the participants. Questions explored when the listening occurred, at what speed, whether simultaneous activities were performed, whether notes were taken, if (and why) the podcast was paused, if (and why) segments were repeated, whether an accent affected listening, and the audio quality. These were based on known effective learning techniques [13], previous podcast research [5-7], and the real-world experiences of the authors.

Protocol

Participants were directed to a dedicated podcast channel and asked to listen to a podcast and respond to a brief questionnaire after listening (Appendix A). Participants were instructed to complete each of the eight podcast surveys immediately after listening to their respective podcasts. They could choose any order in which to listen to the eight podcasts. Participants then received reminder emails for each follow-up survey every one to two weeks up to a maximum of four times.

Analysis

Raw survey data was exported from FluidSurveys in a spreadsheet format, and descriptive statistics were calculated using Microsoft Excel (Microsoft Corp., Redmond, WA, USA).

## Results

Recruitment occurred between September 10, 2017, and December 9, 2017. The study surveys were accessible until March 8, 2018 (a minimum of three months). A total of 390 healthcare professionals formally enrolled in the study, and 241 completed all eight podcast surveys (61.8% study completion rate). Table 2 contrasts the demographics of the participant group that enrolled in the study and the group that completed all surveys. The groups’ demographics were grossly similar.

**Table 2:** Demographics of the METRIQ podcast study participants who enrolled in and completed the study METRIQ, Medical Education Translational Resources: Impact and Quality.

	Variable	Enrolled in the study, n (%)	Completed the study, n (%)
Age	18-24	41 (10.5)	23 (9.5)
	25-34	204 (52.3)	133 (55.2)
	35-44	111 (28.5)	65 (27.0)
	45-54	25 (6.4)	16 (6.6)
	55-65	9 (2.3)	4 (1.6)
	>65	0 (0%)	0
Gender	Female	175 (44.5)	105 (43.6)
	Male	215 (54.7)	135 (56.0)
	Decline to state/other	3 (0.8)	1 (0.4)
Modality of recruitment	Twitter	144 (36.9)	78 (32.4)
	Email or in-person by a study author	99 (25.4)	73 (30.3)
	Facebook	69 (17.7)	47 (19.5)
	METRIQ Blog Study participant	59 (15.1)	44 (18.3)
	Peer referral	35 (9.0)	18 (7.5)
	Medical website or podcast	19 (4.9)	12 (5.0)
	WhatsApp	15 (3.8)	6 (2.5)
Background/level of training	Practicing Physician, Attending, Consultant	111 (27.7)	73 (30.2)
	Resident, House Officer, or Graduate Trainee	79 (19.7)	40 (16.5)
	Medical Student	81 (20.2)	66 (27.3)
	Nurse, Physician Assistant, or Paramedic	121 (30.2)	58 (24.0)
	Nurse, Physician Assistant, or Paramedic Student	4 (1.0)	3 (1.2)
	>1 category	3 (0.7)	2 (0.8)
	Other (Infection Control Practitioner)	2 (0.5)	0 (0)
Home country	Canada	150 (38.5)	122 (50.6)
	United States	118 (30.3)	59 (24.5)
	Europe	67 (17.2)	32 (13.3)
	Australia/New Zealand	23 (5.9)	14 (5.8)
	Africa	19 (4.9)	9 (3.7)
	South America	7 (1.8)	4 (1.7)
	Asia	6 (1.5)	1 (0.4)
Reason for listening to podcasts	To review new literature	298 (75.8)	181 (75.1)
	To learn core material	295 (75.1)	190 (78.8)
	To refresh memory	282 (71.8)	185 (76.8)
	Inspiration	179 (45.5)	109 (45.2)
	Entertainment	160 (40.7)	105 (43.6)
	To feel connected to the community	143 (36.4)	88 (36.5)
	To keep up with the medical terminology	103 (26.2)	57 (23.7)
	For board certification/re-certification review	63 (16.0)	29 (12.0)
	Other	28 (7.1)	19 (7.9)
Podcast involvement	Podcast manager, operator, or owner	38 (9.7)	20 (8.3)
	Not a podcast manager, operator, or owner	352 (90.3)	221 (91.7)
Podcast-listening frequency	Daily	28 (7.2)	34 (14.1)
	Weekly	178 (45.6)	120 (49.8)
	Monthly	102 (26.2)	61 (25.3)
	Yearly	27 (6.9)	16 (6.6)
	Never	28 (7.2)	10 (4.1)

The participants’ listening habits were relatively consistent across the podcasts (Table 3). The average listening speed varied minimally across podcasts from 1.11 to 1.16. Most listened at normal (1x) speed (range: 73.4%-81.9%), and only a minority listened at ≥1.5x speed (range: 13.9%-21.3%).

**Table 3:** Number (percentage) of participants listening to podcasts at each of the podcasts at a range of speeds

	Podcast 1	Podcast 2	Podcast 3	Podcast 4	Podcast 5	Podcast 6	Podcast 7	Podcast 8
Number at 0.5-0.99x (%)	1 (0.3)	1 (0.4)	2 (0.8)	1 (0.4)	2 (0.8)	0 (0.0)	0 (0.0)	1 (0.4)
Number at 1x (%)	253 (81.9)	223 (78.2)	210 (79.8)	200 (78.1)	192 (76.8)	179 (73.4)	191 (78.9)	184 (76.3)
Number at 1.01-1.49x (%)	12 (3.9)	17 (6.0)	12 (4.6)	12 (4.7)	14 (5.6)	13 (5.3)	12 (5.0)	14 (5.8)
Number at 1.5-1.99x (%)	31 (10.0)	30 (10.5)	29 (11.0)	34 (13.3)	32 (12.8)	40 (16.4)	32 (13.2)	31 (12.9)
Number at >2.00x (%)	12 (3.9)	14 (4.9)	10 (3.8)	9 (3.5)	10 (4.0)	12 (4.9)	7 (2.9)	11 (4.6)
Total	309	285	263	256	250	244	242	241

Approximately one-quarter of participants (range 19.6%-28.5%) did not participate in concomitant activities while listening. When concomitant activities were performed, driving (25.9%-31.6%), chores (16.9%-22.5%), and exercise (9.1%-12.4%) were the most common (Table 4).

**Table 4:** Number (percentage) of participants performing concomitant activities while listening to each of the podcasts

Activity performed	Podcast 1	Podcast 2	Podcast 3	Podcast 4	Podcast 5	Podcast 6	Podcast 7	Podcast 8	
None	62 (20.1)	56 (19.6)	66 (25.1)	49 (19.1)	49 (19.6)	63 (25.8)	69 (28.5)	63 (26.1)	
Chores	68 (22.0)	64 (22.5)	50 (19.0)	55 (21.5)	52 (20.8)	42 (17.2)	41 (16.9)	44 (18.3)	
Exercise	28 (9.1)	32 (11.2)	30 (11.4)	30 (11.7)	26 (10.4)	28 (11.5)	30 (12.4)	26 (10.8)	
Driving	80 (25.9)	83 (29.1)	82 (31.2)	86 (33.6)	76 (30.4)	77 (31.6)	70 (28.9)	71 (29.5)	
Other	81 (26.2)	68 (23.9)	54 (20.5)	50 (19.5)	58 (23.2)	47 (19.3)	39 (16.1)	47 (19.5)	
Total	319	303	282	270	261	257	249	251	

Only a small fraction of participants took notes (0.8%-6.1%), but approximately one-third paused the podcast (19.4%-36.6%) with the most common reason being an interruption (13.6%-27.4%) (Table 5). The proportion of listeners that repeated (9.7%-49.3%) a segment of the podcast was quite variable. When portions were repeated, it was most often because the listener felt that they had missed important information (55.0%-68.8%).

**Table 5:** Rationale and number (percentage) of participants pausing or repeating segments while listening to each of the podcasts

Reason cited	Podcast 1	Podcast 2	Podcast 3	Podcast 4	Podcast 5	Podcast 6	Podcast 7	Podcast 8
Pausing								
To stop the flow of new content to allow me to process information	18 (13.3)	19 (15.8)	34 (25.0)	19 (22.9)	10 (11.9)	3 (4.8)	7 (13.2)	7 (11.9)
To facilitate memorization of information before moving on	5 (3.7)	7 (5.8)	20 (14.7)	5 (6.0)	6 (7.1)	3 (4.8)	4 (7.5)	7 (11.9)
To break up the podcast into more digestible segments	9 (6.7)	9 (7.5)	16 (11.8)	3 (3.6)	6 (7.1)	6 (9.7)	4 (7.5)	5 (8.5)
I wasn't able to listen to the entire podcast at once due to interruptions	80 (59.3)	78 (65.0)	58 (42.6)	51 (61.4)	56 (66.6)	41 (66.1)	33 (62.3)	35 (22.0)
Other	23 (17.0)	7 (5.8)	8 (5.9)	6 (7.2)	6 (7.1)	9 (14.5)	5 (9.4)	5 (8.5)
Total	135	120	136	83	84	62	53	59
Repeating								
To reinforce information	10 (14.5)	18 (22.8)	37 (30.3)	16 (27.6)	15 (24.6)	3 (8.3)	4 (11.7)	15 (21.4)
To ensure that I haven't missed important information	23 (33.3)	23 (29.1)	34 (27.9)	17 (29.3)	16 (26.2)	12 (33.3)	9 (26.5)	20 (28.6)
Because I know I have missed important information	32 (46.4)	34 (43.0)	43 (35.3)	22 (37.9)	27 (44.3)	16 (44.4)	17 (50.0)	33 (47.1)
Other	4 (5.8)	4 (5.1)	8 (6.6)	3 (5.2)	3 (4.9)	5 (13.9)	4 (11.7)	2 (2.9)
Total	69	79	122	58	61	36	34	70

## Discussion

Scholars interested in the knowledge translation of new findings in the health sciences must consider the limitations of this modality to maximize effective education for end-users. In this exploration of the podcast-listening behaviors of an interprofessional cohort of health professionals, most participants played podcasts at normal speed and participated in another activity (usually driving) while they were listening. Listeners occasionally stopped the podcasts due to interruptions, while a minority repeated segments for educational purposes. These behaviors were relatively consistent across a sample of eight podcasts.

Similar to previous studies, our participants reported performing simultaneous tasks (e.g., chores, exercise) while listening [5]. Our finding that approximately three-quarters of participants listen at 1x (normal) speed is in line with the self-reported behavior of emergency medicine (EM) residents and clinicians [6,7]. However, fewer of our participants drove relative to the high proportions described in self-report studies (up to 93%) [6].

Our pragmatic approach gleaned several findings that could not be explored using self-report data. For example, the range of participants who paused and repeated sections of each podcast varied, suggesting that there may be differences between podcasts that make the listener more likely to be interrupted or want to review a segment (e.g., poor sound quality, complex content, speaker accent). A common reason cited for pausing or repeating sections was that information was missed. Podcast producers may consider using this information to modify their recordings to incorporate more repetition of key or complex information [14].

Our results reiterate the finding that clinicians are not listening to educational podcasts in ways that align with their most commonly cited reasons for listening: reviewing new literature, learning core material, and refreshing memory (Table 2). Multitasking and the lack of active learning behaviors (pausing, repeating, note-taking) are not in keeping with behaviors that foster a deep understanding of the material [13]. Multitasking in the form of listening and driving simultaneously may increase the listener’s cognitive load, resulting in decreased driving performance and limited retention of podcast content [15]; however, a recent study found similar retention while driving and sitting in an undistracted setting [16]. The effect of exercise on cognitive performance is generally positive but varies with exercise duration, exercise intensity, and participant fitness [17]. Further, roughly 15% of participants listened at speeds ≥1.5x, a point at which there may be a significant drop-off in comprehension of speech [18]. If clinicians desire to listen - and podcasters desire to create podcasts - with a goal of deep learning and retention of the material, they may consider intentionally limiting distractions [19], pausing to enable connections between new material and existing knowledge, and repeating segments at spaced intervals [20]. Our study design did not allow the exploration of the impact of concurrent activities or listening strategies (e.g., variable listening speeds, interruptions, and repeating difficult segments) on knowledge acquisition but would support the further experimental study of these questions.

The strengths of our findings lie in our large and diverse study population and pragmatic approach. The enrolment of a large, diverse population of health professionals to listen to eight different podcasts and complete surveys on each allowed us to describe how podcast listeners utilize medical education podcasts in a real-world context. The results should inform research to determine how podcasts can be maximized for learning.

Limitations

While the number of participants in this study was high, our population was not necessarily representative of the wider body of health professionals. We note that roughly 25% of the cohort completing the study were students. Our goal was not to replicate this population but to recruit clinicians who listen to podcasts regularly as this sample is more relevant to our inquiry than a representative population including those who do not use podcasts. Still, most participants were from developed countries, and a quarter were students. We believe that the discrepancies between self-report and real-world listening habits demonstrate the value of our pragmatic methodology, although it is possible that our results were skewed by participants changing their listening habits to complete the study. Lastly, we lost a significant number of our target population during the study; this was not surprising, given that participation required listening to eight podcasts and completing eight surveys. The drop-off was consistent across the first four podcasts, with fewer participants dropping out on the final four. Fortunately, the demographics of the participants who enrolled in the study were similar to those who completed it, suggesting that this did not have a substantial impact on our results. Finally, a large number of participants did not list their location, restricting any further analysis of the role of the listeners' nationality in listening behavior.

## Conclusions

Educational podcasts are increasingly being utilized by health professionals for continuing education, but how they are being used remains poorly understood. We performed a prospective survey-based study of the listening habits of an interprofessional sample of emergency medicine and critical care clinicians to determine the pragmatic, real-world listening habits of health professionals. Most health professionals who listened to medical education podcasts did so at normal playback speeds and engaged in other activities such as driving or exercise while listening. A minority of listeners paused or repeated segments. This work informs our understanding of the utilization of podcasts by end-users and may guide future research on optimizing podcast use for learning and knowledge translation.

## Appendices

Appendix A: Usage survey

Please respond to these questions in reference specifically to the [insert podcast name] podcast that you were assigned to this week.

1. When did you listen to the podcast?

Morning

Afternoon

Evening

Night

2. What device did you use to listen to the podcast?

Computer

Tablet

Smartphone: Apple

Smartphone: Android

Smartphone: Other

MP3 Player

Car Stereo

Other

3. What was your listening speed?

(e.g., 0.5x would mean that you slow the podcast down to 1/2 speed, while 2.0x would mean that you double the speed of the podcast.)

4. Did you perform any simultaneous unrelated activities?

No

Chores

Exercise

Driving

Other

5. Did you take notes?

Yes

No

6. Did you ever pause the podcast?

Yes

No

7. If yes to the previous question, approximately how many times did you pause it?

8. Did you ever repeat segments of the podcast?

Yes

No

9. If yes to the previous question, how many times did you repeat segments of the podcast?

Podcast critique

1. Foreign accents

Did not impede the listening ability

Difficult but understandable

Indecipherable

N/A

2. Recording quality

High quality

Medium quality

Low quality

3. What was your prior knowledge of the content of the podcast?

None

Introductory

Intermediate

Advanced

Short answer questions

1. What was your key takeaway from the podcast?

2. Was the podcast well designed? Why or why not?

3. Was the content of high quality? Why or why not?

4. Was the podcast credible? Why or why not?
